# Molecular Phylogenetics and Systematics of the Bivalve Family Ostreidae Based on rRNA Sequence-Structure Models and Multilocus Species Tree

**DOI:** 10.1371/journal.pone.0108696

**Published:** 2014-09-24

**Authors:** Daniele Salvi, Armando Macali, Paolo Mariottini

**Affiliations:** 1 CIBIO, Centro de Investigação em Biodiversidade e Recursos Genéticos, InBIO, Universidade do Porto, Campus Agrário de Vairão, Vairão, Portugal; 2 Dipartimento di Scienze, Università “Roma Tre”, Rome, Italy; Institute of Biochemistry and Biology, Germany

## Abstract

The bivalve family Ostreidae has a worldwide distribution and includes species of high economic importance. Phylogenetics and systematic of oysters based on morphology have proved difficult because of their high phenotypic plasticity. In this study we explore the phylogenetic information of the DNA sequence and secondary structure of the nuclear, fast-evolving, ITS2 rRNA and the mitochondrial 16S rRNA genes from the Ostreidae and we implemented a multi-locus framework based on four loci for oyster phylogenetics and systematics. Sequence-structure rRNA models aid sequence alignment and improved accuracy and nodal support of phylogenetic trees. In agreement with previous molecular studies, our phylogenetic results indicate that none of the currently recognized subfamilies, Crassostreinae, Ostreinae, and Lophinae, is monophyletic. Single gene trees based on Maximum likelihood (ML) and Bayesian (BA) methods and on sequence-structure ML were congruent with multilocus trees based on a concatenated (ML and BA) and coalescent based (BA) approaches and consistently supported three main clades: (i) *Crassostrea*, (ii) *Saccostrea*, and (iii) an Ostreinae-Lophinae lineage. Therefore, the subfamily Crassotreinae (including *Crassostrea*), Saccostreinae **subfam. nov.** (including *Saccostrea* and tentatively *Striostrea*) and Ostreinae (including Ostreinae and Lophinae taxa) are recognized. Based on phylogenetic and biogeographical evidence the Asian species of *Crassostrea* from the Pacific Ocean are assigned to *Magallana* **gen. nov.**, whereas an integrative taxonomic revision is required for the genera *Ostrea* and *Dendostrea*. This study pointed out the suitability of the ITS2 marker for DNA barcoding of oyster and the relevance of using sequence-structure rRNA models and features of the ITS2 folding in molecular phylogenetics and taxonomy. The multilocus approach allowed inferring a robust phylogeny of Ostreidae providing a broad molecular perspective on their systematics.

## Introduction

The bivalve family Ostreidae (oysters) includes about 75 species distributed worldwide along the coast of all continents with the exception of Antarctica and some oceanic islands [1]. Oysters are sessile filter-feeders and play an important role in marine ecosystems through the mitigation of the excess of sediment, nutrients, and algae in estuarine and intertidal waters. Several species are of economic importance, being among the most highly produced mollusc species in the world by aquaculture industry (http://www.fao.org/fishery/en). Since their economic significance and important role in marine ecosystems, oysters are among the most studied groups of marine bivalves [2]. Despite that, phylogenetic relationships of oysters are not yet well understood and species classification and identification remain difficult [1].

The current classifications of living oysters [1], [3] are mainly based on the comprehensive study of Harry [4], who utilized both shell and soft-part morphology and proposed the current arrangement of the family Ostreidae in the three subfamilies Crassotreinae, Ostreinae and Lophinae. However, oyster shell morphology shows a high degree of phenotypic plasticity, with environmental factors such as the nature of the substrate and/or the tidal regime strongly influencing valve morphology [5], [6]. As within species variation in shell morphology is extensive and many sympatric species converge to similar ecophenotypic variants, phylogenetic and taxonomic analyses of oyster based on morphological characters are prone to errors [1], [7]–[11].

In the last decade, the analysis of DNA sequence data has improved our understanding of oyster relationships [12]–[17] and provided suitable molecular tools for species identification [18]; [11], [19]–[21]. Some species with a wide distribution has revealed to be a complex of different species sharing a similar morphology (e.g. [8], [13]) many other to be a single taxon introduced across the oceans [22], [23]. Most research have focused on relationships and identification of species of economic importance with a narrow taxonomic (within-genus) and geographic (regional-scale) focus. Those studies that have included more than two genera have systematically found incongruent results with morphology-based phylogeny and taxonomy of oysters, in some cases challenging the traditional view that oyster subfamilies are monophyletic [8], [11], [12], [24]–[26].

To date, molecular phylogenetic inferences of oyster relationships have mostly relied on DNA sequences data from a single locus. Fragments of ribosomal genes from either the mitochondrial or the nuclear large subunits (16S and 28S rRNA genes, respectively) have been employed [8], [12], [23], [24]. On the other hand, the availability of data from fast-evolving genes is limited to mitochondrial sequences of the conventional ‘barcoding gene’ *cytochrome c oxidase I* (COI) and other protein coding genes [11], [26] for *Crassostrea*, *Saccostrea*, and *Ostrea* species.

Molecular data from fast-evolving nuclear genes, and the implementation of a multilocus approach are needed to obtain a robust phylogenetic hypothesis of oysters relationships that can be used as a basis for their systematics. The nuclear ribosomal internal transcribed spacer (ITS2) has repeatedly proven to be a valuable marker for bivalves phylogenetics and taxonomy, especially when the information from both the sequence and secondary structure are exploited (see e.g. Pectinidae [27]; Veneridae [28]). Including RNA secondary structures improves accuracy and robustness in reconstructing phylogenetic trees [29]. Moreover, conserved sequence-structure features of the ITS2 rRNA gene, such as stem-loops domains and compensatory base changes (CBCs), have shown to be diagnostic of a wide-range of bivalves taxonomic groups providing further support for their monophyly and for their molecular diagnosis ([28]; see also [30]).

In this study, we analysed in a phylogenetic framework the primary sequence and secondary structure information from the nuclear ITS2 and mitochondrial 16S ribosomal genes from the Ostreidae. We employed both traditional phylogenetic methods using sequence data and recently developed tools to simultaneously infer alignments and phylogenies based on the primary sequence and the secondary structure information [31], [32], [33]. We analysed conserved structural elements of the ITS2 folding to pinpoint sequence-structure sinapomorphies supporting phylogenetic relationships between clades as well as sequence-structure autapomorphies distinctive to given terminal groups, valuable for their molecular diagnosis. In addition to single-locus phylogenies we carried out multilocus phylogenetic inferences using the combined information of the mitochondrial 16S, COI, and the nuclear ITS2 and 28S gene sequence datasets and we use the resulting species tree as a guide for molecular systematics of Ostreidae.

The main aims of this study were (i) to assess the phylogenetic and taxonomic information of sequence-structure of the 16S and ITS2 rRNA genes; (ii) to assess the potential efficacy of the genes so far sequenced in oysters as DNA barcode tools for taxonomic identification; (iii) to infer a robust phylogeny of Ostreidae based on combined sequence data from mitochondrial and nuclear loci; and (iv) to provide a molecular perspective on the systematic of oysters.

## Methods

### Dataset, DNA extraction, amplification and sequencing

Sequences from the ITS2 and 16S ribosomal DNA of 34 Ostreidae species, the Gryphaeidae *Hyotissa hyotis* and *Neopycnodonte cochlear*, the Pteridae *Pinctada imbricata* and the Mytilidae *Mytilus edulis* were either obtained from alcohol-preserved specimens or retrieved from the GenBank and employed in the molecular analyses. Details on sample data, along with Genbank accession numbers, vouchers numbers and repository museum, are provided in Table 1. Additionally to ITS2 and 16S dataset, for multilocus phylogenetic analyses we assembled a cytochrome oxidase I (COI) and a 28S rDNA (28S) sequence datasets. We selected COI and 28S sequences from 21 and 24 Ostreidae species, respectively, and the outgroup taxa *H. hyotis*, *H. imbricata* and *N. cochlear*, based on preliminary phylogenetic analyses on 783 sequences of COI and 42 sequences of 28S downloaded from Genbank (see Figure S1 and S2 for more details).

**Table 1:** Species and sample data, along with Genbank accession numbers, of ITS2 and16S rRNAs gene sequences (Genbank accession numbers of COI and28S rRNA sequences are reported in Figure S1 and S2, respectively).

Family	Subfamily	Species	Individual code	Acronym	16S GenBank Number	ITS2 GenBank Number	Specimen voucher Number**
OSTREIDAE	Crassostreinae	*Crassostrea ariakensis*	*Crassostrea ariakensis* (Car-1)	Car-1	-	EU072457*	
			*Crassostrea ariakensis* (Car-2)	Car-2	-	EU252081*	
			*Crassostrea ariakensis* (Car-3)	Car-3	-	FJ356685*	
			*Crassostrea ariakensis* (Car-4)	Car-4	FJ841964*	-	
			*Crassostrea ariakensis* (Car-5)	Car-5	AY160757*	-	
		*Crassostrea brasiliana*	*Crassostrea brasiliana (Cbr-1)*	Cbr-1	-	FJ478044*	
			*Crassostrea brasiliana (Cbr-2)*	Cbr-2	-	FJ544300*	
			*Crassostrea brasiliana (Cbr-3)*	Cbr-3	FJ478029*	-	
		*Crassostrea gasar*	*Crassostrea gasar* (Cga-1)	Cga-1	-	FJ544308*	
			*Crassostrea gasar* (Cga-2)	Cga-2	EF473271*	-	
		*Crassostrea gigas*	*Crassostrea gigas (Cgi-A)*	Cgi-A	-	LM993864	BAU01706
			*Crassostrea gigas (Cgi-B)*	Cgi-B	-	LM993865	BAU01707
			*Crassostrea gigas (Cgi-C)*	Cgi-C	-	LM993866	BAU01708
			*Crassostrea gigas (Cgi-D)*	Cgi-D	-	LM993867	BAU01709
			*Crassostrea gigas (Cgi-1)*	Cgi-1	FJ478036*	-	
			*Crassostrea gigas (Cgi-2)*	Cgi-2	AJ553903*	-	
		*Crassostrea hongkongensis*	*Crassostrea hongkongensis (Cho-1)*	Cho-1	-	GU338879*	
			*Crassostrea hongkongensis (Cho-2)*	Cho-2	FJ841963*	-	
		*Crassostrea nippona*	*Crassostrea nippona (Cni-1)*	Cni-1	-	FJ356681*	
			*Crassostrea nippona (Cni-2)*	Cni-2	-	EU072459*	
			*Crassostrea nippona (Cni-3)*	Cni-3	-	EU252080*	
			*Crassostrea nippona (Cni-4)*	Cni-4	HM015198*	-	
		*Crassostrea rhizophorae*	*Crassostrea rhizophorae (Crh-1)*	Crh-1	-	FJ478039*	
			*Crassostrea rhizophorae* (Crh-2)	Crh-2	FJ478032*	-	
			*Crassostrea rhizophorae* (Crh-3)	Crh-3	AJ312938*	-	
		*Crassostrea virginica*	*Crassostrea virginica* (Cvi-1)	Cvi-1	-	EU072460*	
			*Crassostrea virginica* (Cvi-2)	Cvi-2	AY905542*	-	
		*Saccostrea cucullata*	*Saccostrea cucullata* (Scu-1)	Scu-1	AF458901*	-	
		*Saccostrea echinata*	*Saccostrea echinata* (Sec-1)	Sec-1	AF463493*	-	
		*Saccostrea glomerata*	*Saccostrea glomerata* (Sgl-1)	Sgl-1	AF353101*	-	
		*Saccostrea glomerata*	*Saccostrea glomerata* (Sgl-2)	Sgl-2	AF353100*	-	
		*Saccostrea palmula*	*Saccostrea palmula* (Spa-1)	Spa-1	AF768501*	-	
		*Saccostrea palmula*	*Saccostrea palmula* (Spa-2)	Spa-2	-	-	
		*Saccostrea kegaki*	*Saccostrea kegaki* (Ske-1)	Ske-1	-	EU072464*	
		*Saccostrea scyphophilla*	*Saccostrea scyphophilla (Ssc-A)*	Ssc-A	LM993882	LM993868	BAU01710
			*Saccostrea scyphophilla (Ssc-B)*	Ssc-B	LM993883	LM993869	BAU01711
			*Saccostrea scyphophilla (Ssc-3)*	Ssc-3	HQ660993	-	
		*Striostrea circumpicta*	*Striostrea circumpicta (Sci-1)*	Sci-1	-	EU072462*	
	Lophinae	*Alectryonella plicatula*	*Alectryonella plicatula* (Apl-1)	Apl-1	AF052072*	-	
		*Dendostrea crenulifera*	*Dendostrea crenulifera* (Dcr-1)	Dcr-1	EU815985*	-	
		*Dendostrea folium*	*Dendostrea folium* (Dfo-A)	Dfo-A	LM993884	LM993870	BAU01712
			*Dendostrea folium* (Dfo-B)	Dfo-B	LM993885	LM993871	BAU01713
			*Dendostrea folium* (Dfo-1)	Dfo-1	AF052069*	-	
		*Dendrostrea frons*	*Dendrostrea frons* (Dfr-1)	Dfr-1	AF052070*	-	
		*Dendrostrea rosea*	*Dendrostrea rosea* (Dro-1)	Dro-1	EF122381*	-	
		*Lopha cristagalli*	*Lopha cristagalli* (Lcr-1)	Lcr-1	AF052066*	-	
	Ostreinae	*Ostrea algoensis*	*Ostrea algoensis* (Oal-1)	Oal-1	AF052062*	-	
		*Ostrea angasi*	*Ostrea angasi* (Oan-1)	Oan-1	AF052063*	-	
			*Ostrea angasi* (Oan-2)	Oan-2	AF540594*	-	
		*Ostrea auporia*	*Ostrea auporia*(Oau-1)	Oau-1	AF052064*	-	
		*Ostrea chilensis*	*Ostrea chilensis* (Och-1)	Och-1	AF052065*	-	
		*Ostrea conchaphila*	*Ostrea conchaphila (Oco-1)*	Oco-1	-	EF035117*	
			*Ostrea conchaphila (Oco-2)*	Oco-2	-	EF035120*	
			*Ostrea conchaphila (Oco-3)*	Oco-3	AF052071*	-	
			*Ostrea conchaphila (Oco-4)*	Oco-4	FJ768527*	-	
		*Ostrea denselamellosa*	*Ostrea denselamellosa (Ode-1)*	Ode-1	-	FJ356689*	
			*Ostrea denselamellosa (Ode-2)*	Ode-2	-	EU072463*	
			*Ostrea denselamellosa (Ode-3)*	Ode-3	-	EU252082*	
			*Ostrea denselamellosa (Ode-4)*	Ode-4	AF052067*	-	
			*Ostrea denselamellosa (Ode-5)*	Ode-5	HM015199*	-	
		*Ostrea edulis*	*Ostrea edulis (Oed-A)*	Oed-A	-	LM993872	BAU01714
			*Ostrea edulis (Oed-B)*	Oed-B	-	LM993873	BAU01715
			*Ostrea edulis (Oed-C)*	Oed-C	-	LM993874	BAU01716
			*Ostrea edulis (Oed-D)*	Oed-D	-	LM993875	BAU01717
			*Ostrea edulis (Oed-1)*	Oed-1	JF274008*	-	
			*Ostrea edulis (Oed-2)*	Oed-2	DQ280032*	-	
		*Ostrea equestris*	*Ostrea equestris* (Oeq-1)	Oeq-1	FJ768588*	-	
		*Ostrea lurida*	*Ostrea lurida* (Olu-1)	Olu-1	AF052073*	-	
		*Ostrea pulcheana*	*Ostrea pulcheana* (Opu-1)	Opu-1	DQ640402*	-	
		*Ostrea spreta*	*Ostrea spreta* (Osp1)	Osp1	DQ640402*	-	
		*Cryptostrea permollis*	*Cryptostrea permolli* (Cpe-1)	Cpe-1	AF052075*	-	
		*Teskeyostrea weberi*	*Teskeyostrea weberi* (Twe-1)	Twe-1	AY376601*	-	
GRYPHAEIDAE	Gryphaeinae	*Hyotissa hyotis*	*Hyotissa hyotis* (Hhy-1)	Hhy-1	-	EU072465*	
			*Hyotissa hyotis* (Hhy-A)	Hhy-A	LM993886	LM993876	BAU01718
			*Hyotissa hyotis* (Hhy-B)	Hhy-B	LM993887	LM993877	BAU01719
			*Hyotissa hyotis* (Hhy-2)	Hhy-2	AY376599*	-	
			*Hyotissa hyotis* (Hhy-3)	Hhy-3	GQ166564*	-	
		*Neopycnodonte cochlear*	*Neopycnodonte cochlear* (Nco-A)	Nco-A	-	LM993878	BAU01720
			*Neopycnodonte cochlear* (Nco-B)	Nco-B	-	LM993879	BAU01721
			*Neopycnodonte cochlear* (Nco-C)	Nco-C	-	LM993880	BAU01722
			*Neopycnodonte cochlear* (Nco-1)	Nco-1	JF496758*	-	
PTERIDAE		*Pinctada imbricata*	*Pinctada imbricata* (Pim-1)	Pim-1	-	LM993881	BAU01723
			*Pinctada imbricata* (Pim-2)	Pim-2	HQ329423*	-	
MYTILIDAE	Mytilinae	*Mytilus edulis*	*Mytilus edulis* (Med-1)	Med-1	-	AY695798*	

Taxonomic designation follows the World Register of Marine Species [3].

*sequences obtained from Genbank;

**specimens deposited at MZUR (Museo Zoologico Università di Roma), Università La Sapienza.

Genomic DNA was extracted from dissected foot of specimens by using the “DNeasy Blood and Tissue Kit” (Qiagen, Hilden, Germany). Polymerase chain reaction (PCR) amplification were performed using the primers ITS2-3d and ITS2-4r [34] targeting the entire ITS2 region, and the primers16Sar-L and 16Sbr-H [35] for the partial 16S fragment (PCR cycling: 3 min at 94°C, 35 cycles of 30 s at 94°C, 30 s at 50–55°C, 90 s at 72°C, 6 min at 72°C for final extension). It is worth mentioning that, despite some oysters show a split of the 16S rNA gene into two segments, e.g. in the Asian *Crassostrea*, *C. virginica*, *Saccostrea mordax*, and *Ostrea edulis* [26], [36], our target 16S fragment (3′ half portion of the 16S rRNA gene corresponding to domains IV and V) is entirely located in the second segment. Therefore, this peculiar characteristic of some oysters did not affect either the PCRs or downstream analyses (secondary structure and phylogenetic analyses). Amplicons were either sequenced directly or cloned by means of the TA Cloning kit (Invitrogen) and the pGEM-T easy Vector System (Promega). Purification and sequencing of Plasmid DNA from positive clones and PCR products were carried out by an external service (Genechron, Rome). The ITS2 delimitation was carried out in accordance with the annotation of published oysters ITS2 sequences and confirmed by examining the tailing 3′ and 5′ parts of the ribosomal 5.8S and 28S rRNA gene sequences.

### Secondary structure modeling and sequence alignment

The ITS2 and 16S secondary structures were obtained contrasting several candidate low free energy folding models calculated using RNA structure 5.5 [37] against secondary structure models proposed for molluscs in previous studies [27], [28], [38], [39]. A further attempt to predict ITS2 secondary structure based on comparisons with available structures from ITS2-Database [40] returned no blast hit.

ITS2 and 16S multiple sequence alignments were performed while simultaneously considering the secondary structure of each sequence. For the ITS2 dataset, we used the Clustal W algorithm based on a sequence-structure scoring matrix specific to eukaryotic ITS2 implemented in 4SALE 1.7 [41], [42]. Alignment of 16S sequences was performed using ClustalX 2.0 [43] and progressively optimized according to secondary structure homology. For both ITS2 and 16S we produced two set of alignments: multiple sequences alignments and multiple sequence-structure alignments which included individual sequences and their secondary structures translated in the Vienna format. The COI and 28S sequence alignments were performed using ClustalX. Conserved and variable sites for each dataset as well as intra- and inter-specific Kimura 2-parameter distances (gaps threated with the complete deletion option) were calculated using MEGA 6 [44].

### Phylogenetic analyses based on primary sequence and secondary structure information

Phylogenetic analyses based on solely ITS2 and 16S primary sequence information (sequence alignments) were carried out using Maximum likelihood (ML) and Bayesian (BA) approaches, with four outgroup combinations: (i) *P. imbricata*, (ii) *M. edulis*, (iii) *P. imbricata* and *M. edulis*, (iv) *H. hyotis* and *N. cochlear*. ML analyses were conducted in TREEFINDER v. October 2008 [45] implementing the optimal models of nucleotide substitution selected by TREEFINDER under the corrected Akaike's Information Criterion (ITS2: GTR+G; 16S: TVM+G). We performed Global tree Searches using 100 random start trees generated through equidistant random walks of random nearest-neighbour-interchanges (NNI) starting from the centre trees obtained by simple ML searches. Nodal support was calculated using 1000 bootstrap replicates (BP). BA analyses were performed in MrBayes 3.2 [46] using the same substitution models as for ML analyses. Two independent Markov chain Monte Carlo (MCMC) analyses were run in parallel for 10^7^ generations, sampling every 1000 generations. MCMC chains convergence was verified by average standard deviation of split frequencies values below 0.0028. The 7,500 trees (75%) sampled after burn-in were used to assess posterior probabilities for nodal support (BPP).

ITS2 and 16S sequence-structure alignments were used to infer phylogenetic trees simultaneously on primary sequence and secondary structure information using the gryphaeid taxa *H. hyotis* and *N. cochlear* as as outgroups. We implemented a ML approach based on a combined model of sequence-structure evolution within the R framework (R Development Core Team, 2011) using the R library ‘Phangorn’ [32]. First, we use 4SALE to translate the sequence-structure alignments in 12 states alignments combining the 4 nucleotide states (A, G, C, U) with the three structural states (unpaired, paired left, paired right: “.”, “(“,”)”, respectively). Then, we estimated a GTR+I+G model of rRNA sequence-structure evolution that we used for the ML tree searches. The robustness of the phylogenetic trees was tested by 1000 bootstrap replicates.

Multilocus phylogenetic inferences were carried out using the combined information from 16S, COI, ITS2 and 28S data. First, we carried out concatenated ML and BI analyses on the mitochondrial (16S+COI) and the nuclear (ITS2+28S) dataset separately, and then on the combined 16S+COI+ITS2+28S dataset. ML analyses were performed in TREEFINDER using the optimal model of nucleotide substitution for each gene (16S: TVM+G; COI: HKY+G; ITS2: GTR+G; 28S: TN+G) and following the Global tree Search procedure described above. Multilocus BA analyses were performed in BEAST 1.7.5 [47] implementing linked tree models for the mitochondrial genes (16S and COI) and for the ribosomal nuclear genes (ITS2 and 28S), because they are genetically linked. Substitution models and relaxed clock models were unlinked across all markers. We specified a Yule process of speciation as tree prior and a random starting tree. All BEAST analyses were run twice, with two independent runs, with 3×10^7^ iterations per run, sampling every 3,000 steps. Results of the runs were analysed, combined and summarized with Tracer v1.5 [48] LogCombiner and TreeAnnotator (both in the BEAST package). Consensus tree representing the posterior distribution were visualised and edited in FigTree v1.4 [49].

Finally, we further evaluated the phylogenetic relationships among oysters by using the multi-species coalescent-based method (species-tree) implemented in the *BEAST extension (Heled and Drummond 2010) of the BEAST 1.7.5 package. The species-tree analysis was performed using linked tree models for the mitochondrial genes and for the ribosomal nuclear genes, unlinked substitution model parameters and clock models across loci and a Yule process of speciation as tree prior. *BEAST runs were 3×10^8^ iterations long, with a sampling frequency of 30,000 steps.

### Nomenclatural Acts

The electronic edition of this article conforms to the requirements of the amended International Code of Zoological Nomenclature, and hence the new names contained herein are available under that Code from the electronic edition of this article. This published work and the nomenclatural acts it contains have been registered in ZooBank, the online registration system for the ICZN. The ZooBank LSIDs (Life Science Identifiers) can be resolved and the associated information viewed through any standard web browser by appending the LSID to the prefix “http://zoobank.org/”. The LSID for this publication is: urn:lsid:zoobank.org:pub:C0247395-1FD2-4A4D-9957-6F855D508C6B. The electronic edition of this work was published in a journal with an ISSN, and has been archived and is available from the following digital repositories: PubMed Central, LOCKSS.

## Results and Discussion

### ITS2, 16S, COI and 28S sequence variation and DNA barcoding performance

The oyster ITS2 rRNA sequences ranged in length from 401 (*Ostrea conchaphila* and *Striostrea circumpicta*) to 545 (*Crassostrea gigas*) base pairs (bp). Intra-individual variation of the ITS2 sequences was not observed in any species. Multiple ITS2 sequence alignment resulted in a total of 883 nucleotide positions including indels, among which 419 positions were variable. The length of 16S sequences ranged from 507 (*Crassostrea rhizophorae*) to 520 (*Saccostrea scyphophilla*) bp. The 16S alignment comprised a total of 565 nucleotide positions including indels, among which 297 positions were variable. The COI alignment was 620 bp long and required no gaps. Among the 326 variable sites, 85 were in the first, 37 in the second, and 204 in the third codon position. The 28S alignment was 956 bp long with 299 variable positions.

Intraspecific genetic distance of ostreid taxa ranged from 0 to 0.37/1.17/2.26/2.97% for the 28S, ITS2, COI, and 16S dataset respectively (K2p distance). The genetic distance observed between the species *Crassostrea brasiliana* and *C. gasar* clearly fell within this range (ITS2/16S/COI K2p distance: 0/0.36/0.44%), suggesting that these two species should be synonymized as *C. gasar* as proposed by [20]. Once accounted for this case, the minimum interspecific genetic distance between ITS2 sequences of ostreid were 2.75%, between *C. ariakensis* and *C. hongkongensis*, and in remaining cases >6%, supporting the suitability of the ITS2 as DNA barcode marker for ostreids (see also [21]). The performance of 16S and COI for DNA barcoding of ostreids has been previously discussed by [11], [50]. Our results are in agreement with this study. Both markers did not show a barcoding gap between intraspecific and interspecific level of genetic differentiation, although many of the overlaps could be discussed in the framework of taxonomic revisions. Species pairs such as *Ostrea edulis/O. angasi*, *Ostrea equestris/O. aupouria*, and *Cryptostrea permollis/Ostrea puelchana* showed very low genetic distances at both markers (16S and COI K2p distance <0.73% and <3.66%, respectively). Very low 16S genetic distance values (K2p distance <0.36%) were observed between the species *Ostrea auporia/O. equestris/O. spreta*, and *Ostrea conchaphila/O. lurida*. Once accounting for these cases, the COI showed a barcoding gap between intraspecific (K2p distance <3.66%) and interspecific differentiation (K2p distance>12.45%). On the other hand, for the 16S dataset we observed several interspecific distance values (within the genera *Crassostrea*, *Saccostrea*, and *Ostrea* and among species belonging to *Alectryonella*, *Dendrostrea*, and *Lophia*) in a twilight zone between the intraspecific and interspecific level (K2p distance ranging from 1.46% to 2.96%). A complete lack of barcoding gap was observed in the 28S dataset showing several interspecific genetic distance within the genera *Crassostrea*, *Saccostrea*, and *Ostrea* below the 0.5%. Therefore, based on our results and previous studies we recommend the nuclear ITS2 and the mitochondrial COI as molecular markers for DNA barcoding of ostreids.

### 16S and ITS2 rRNA secondary structure of Ostreidae

The derived secondary structure of the 3′ half portion of the Ostreidae 16S rRNA gene examined in the present work (corresponding to domains IV and V) conforms to the canonical architecture proposed for eukaryotes [51], [52] and molluscs [37] and does not allow any significant structural discrimination among the taxa analysed. Both domains IV and V show a high conservation in folding when compared to the secondary structures from other bivalves [27]. Stems, bulges and loops in the secondary structure derived from the oyster sequences analysed were structurally fairly conserved providing a useful guide for sequence alignment.

**Figure 1:**
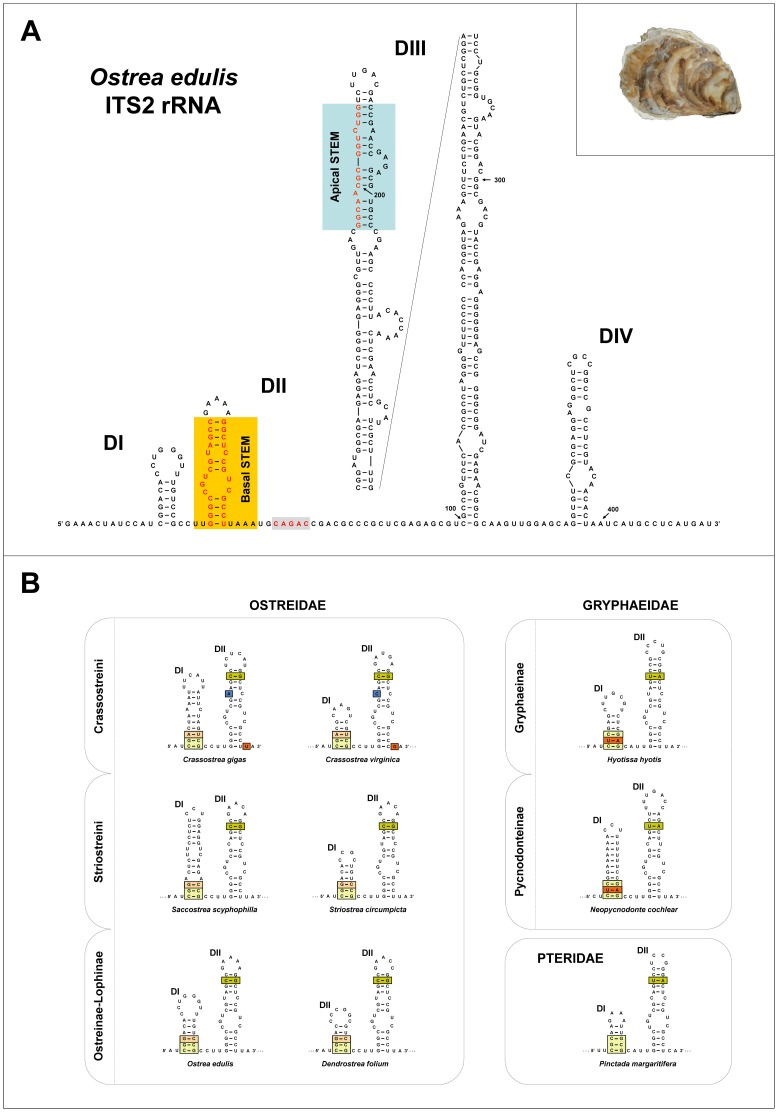
ITS2 secondary structure models for Ostreidae, Gryphaeidae and Pteridae. (**A**) ITS2 secondary structure model for Ostreidae showing the typical four domain folding (exemplified in the type-species *Ostrea edulis*). DI–IV, stem-loops domains. Conserved sequences are boxed, see text for definition. (**B**) secondary structure models of Domain I and II. Conserved sequences are boxed with different colors indicating differences among taxa. See text for explanation. Acronyms are: Cgi, *Crassostrea gigas*; Cvi, *Crassostrea virginica*; Hhy, *Hyotissa hyotis*; Oed, *Ostrea edulis*; Pma, *Pinctada imbricate*; Ssc, *Saccostrea scyphophilla*.

The typical oyster ITS2 rRNA folding along with conserved secondary structure elements across Bivalvia is represented in Figure 1A. As described for eukaryotes [53], [54] the common derived Ostreidae ITS2 rRNA structure is generally organized in four-five stems, defined as DI–V (see [27] for secondary structure nomenclature). The DI–III domains are always identifiable in terms of sequence/structure and position; particularly, DII is equivalent to the Basal STEM described in Bivalvia [28] and DIII, albeit variable in sequence length, is easily identifiable since invariantly shows the Apical STEM consensus sequence [28]. The putative CAGAC motif, consensus of the metazoan 8S rRNA cleavage site [55], [56] was present within the single strand region located between DII and DIII in all taxa analysed (Fig. 1A). Overall, the ITS2 rRNA folding is rather conserved across oysters, yet specific phylogenetic groups showed the presence of diagnostic sequence-structure characters which are discussed in the following section.

### Sequence-structure phylogenetic and systematic of the family Ostreidae

Within the superfamily Ostroidea, the family Gryphaeidae (honeycomb oysters) resulted to be separated from Ostreidae by morphology and molecular analysis (e.g. [1], [12], [57]–[59]). Both the ITS2 and 16S rRNA markers confirm the familial separation. Indeed, all the phylogenetic analyses (ML, BI, seq-str ML) performed on the ITS2 and 16S sequences using nonostreoidean outgroups (*P. imbricata* and *M. edulis*) showed the two families as reciprocally monophyletic with high support (BP≥80, BPP≥0.98; results not shown). Interestingly, although in both families the ITS2 RNA sequence is organized in four to five helix domains (DI–V) of secondary structure, some complementary base-pairing features of the DI and Basal STEM (Fig. 1B), as well as in the Apical STEM of DIII (not shown), can be considered as familial specific land-marks. In particular, compensatory base changes (CBCs) are present in three highly conserved RNA double helix motifs: (i) in the basal portion of DI the Ostreidae triplet 5′-CGG/CCG-3′, changes to 5′-CUC/GAG-3′ in Gryphaeidae; (ii) in the upper portion the Basal STEM the Ostreidae quadruplet 5′-AGCC/GGCU-3′ changes to 5′-AGUC/GACU-3′ in Gryphaeidae; and (iii) in the Apical STEM the Ostreidae sequence is 5′-GGCA^A^ CGYGGUCUGC-3′,while in Gryphaeidae is 5′-AGCA^A^ UGCGGUCUGC-3′. Conserved sequence-structure motifs in the ITS2 secondary structure are rare or unique features likely to be of single evolutionary origin. Therefore the three alternative sequence-structure features pointed out in the families Gryphaeidae and Ostroidea support their reciprocal monophyly and provide a useful tool for their molecular diagnosis.

**Figure 2:**
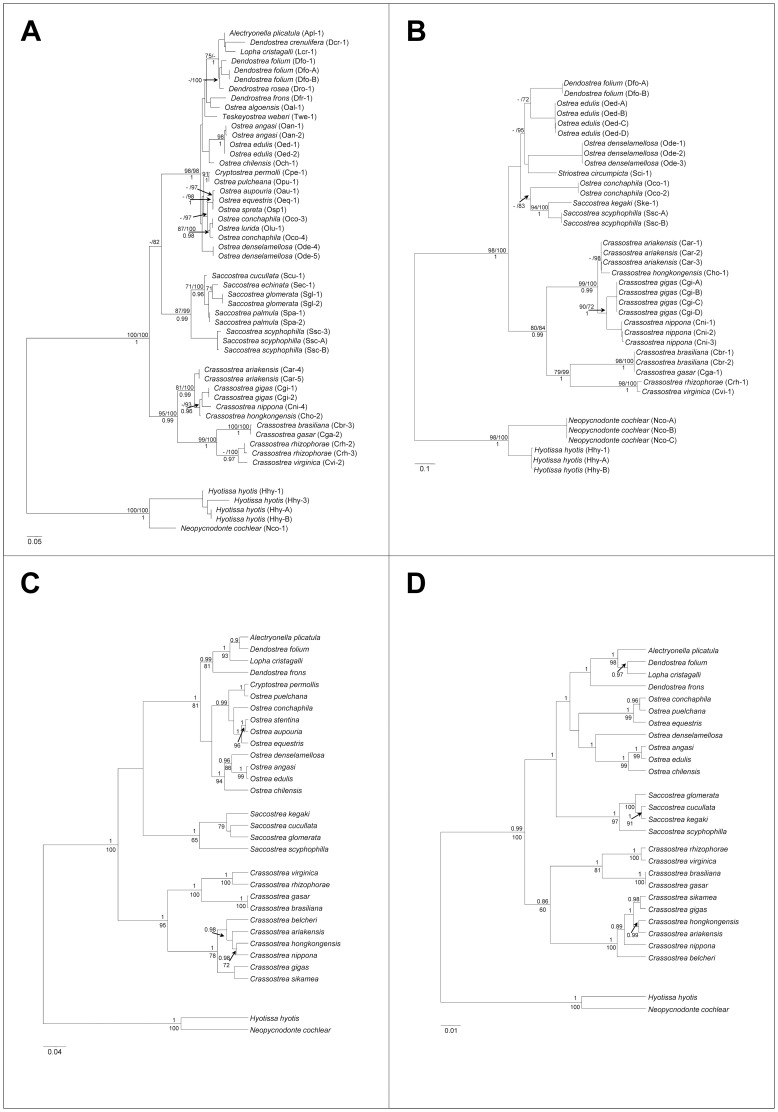
Phylogeny of the Ostreidae derived from mitochondrial 16S rRNA and COI, and nuclear ITS2 rRNA and 28S rRNA gene sequence datasets using *Hyotissa hyotis* and *Neopycnodonte cochlear* (Gryphaeidae) as outgroup. (**A–B**) Maximum-Likelihood phylogenetic trees based on the 16S rRNA (**A**) and ITS2 rRNA (**B**) gene fragments. Above the nodes are reported bootstrap values (BP) ≥70 of the Maximum-Likelihood phylogenetic analyses based on primary sequence (ML) and on sequence-structure alignments (MLseq-str) (BP_ML_/BP_MLseq-str_); below the nodes are reported Bayesian posterior probabilities values (BPP) ≥0.9 of the Bayesian phylogenetic analyses. See Table 1 for details on sequence used and acronyms (BA). (**C–D**) Bayesian phylogenetic trees based on the 16S+COI (**C**) and ITS2+28S (**D**) combined sequence datasets. Above the nodes are reported BPP values ≥0.9 of the Bayesian analyses; below the nodes are reported BP≥70 of the Maximum-Likelihood analyses. See Table 1 and Figure S1 and S2 for details on sequence used and acronyms.

**Figure 3:**
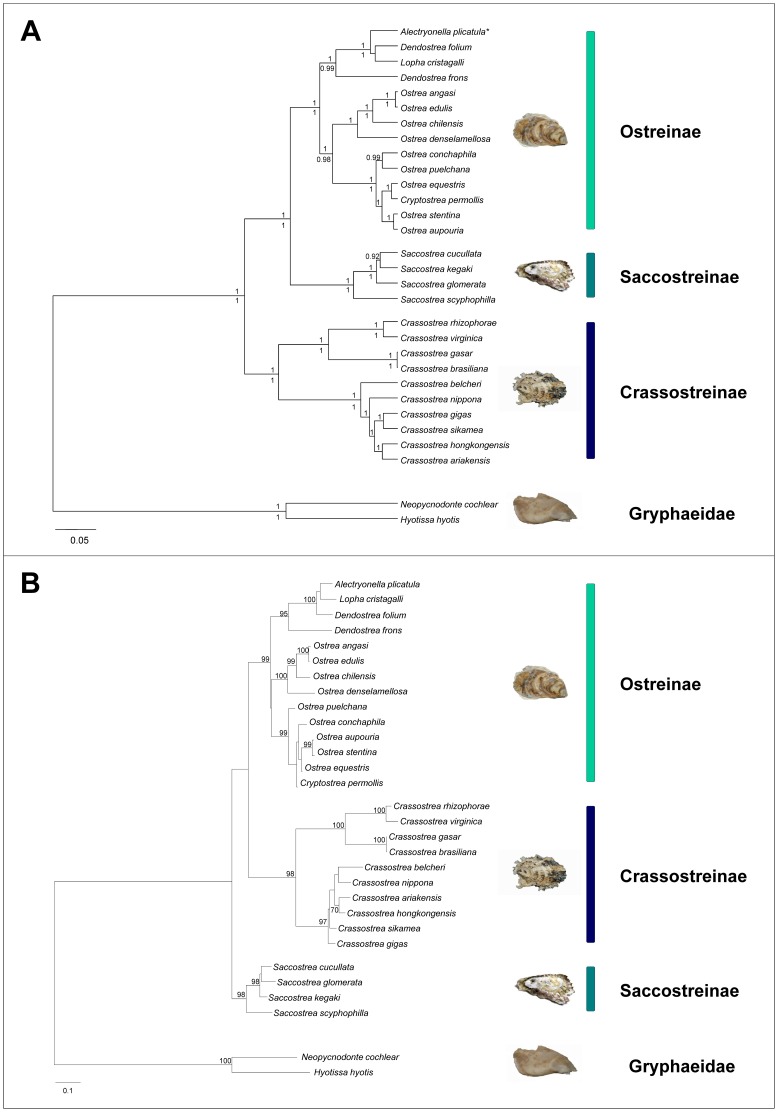
Phylogenetic and systematic relationships of the Ostreidae species derived from Bayesian and Maximum-likelihood analyses combining 16S rRNA, COI, ITS2 rRNA, and 28S rRNA gene sequence datasets using *Hyotissa hyotis* and *Neopycnodonte cochlear* (Gryphaeidae) as outgroup. (**A**) Bayesian tree based on the concatenated analysis of the gene sequence datasets with Bayesian posterior probabilities (BPP) ≥0.9 reported above the nodes. Below the nodes are reported the BPP ≥0.9 of the Bayesian analyses performed implementing a multi-species coalescent model in the *BEAST software. (**B**) Maximum-likelihood tree based on the concatenated analysis of the gene sequence datasets with bootstrap values ≥70 reported in correspondence of the nodes.

Our study analysed for the first time mitochondrial and nuclear DNA sequences data for most of the genera of the family Ostreidae in a phylogenetic framework, thus providing a robust hypothesis of ostreid phylogeny as well as a test of their current systematic. According to [4] and [3] the Ostreidae currently comprises three subfamilies: (i) Crassostreinae Scarlato & Starobogatov, 1979, including the genera *Crassostrea* Sacco, 1897, *Saccostrea* Dollfus & Dautzenberg, 1920, *Striostrea* Vialov, 1936, *Talonostrea* Li & Qi, 1994. The genera *Crassostrea, Saccostrea* and *Striostrea* were represented in our dataset; (ii) Lophinae Vialov, 1936, including the genera *Lopha* Röding, 1798, *Alectryonella* Sacco, 1897, *Dendostrea* Swainson, 1835, and *Myrakeena* Harry, 1985 (the latter genus tentatively placed in Ostreinae by [1]). The genera *Lopha, Alectryonella* and *Dendostrea* were included in our dataset; (iii) Ostreinae Rafinesque, 1815, including the genera *Ostrea* Linnaeus, 1758, *Booneostrea* Harry, 1985, *Nanostrea* Harry, 1985, *Pustulostrea* Harry, 1985, *Teskeyostrea* Harry, 1985, *Undulostrea* Harry, 1985. Huber [1] considers *Ostreola* Monterosato, 1884, *Cryptostrea* Harry, 1985 and *Planostrea* Harry, 1985 as valid genera rather than subgenera or synonyms of *Ostrea*. In our dataset the genera *Ostrea* and *Teskeyostrea* and the putative genera *Ostreola* and *Cryptostrea* were represented. The phylogenetic relationships as inferred from our analyses suggest that none of the currently recognized subfamilies is monophyletic and support a different systematic arrangement, with three major clades corresponding to (i) *Crassostrea* (ii) *Saccostrea*; and (iii) an Ostreinae-Lophinae lineage (Figs. 2 and 3). These three lineages are supported by all the phylogenetic analyses either based on single genes (16S, COI, 28S) or on multilocus data (mitochondrial, nuclear and their combination), independently from the method used (ML, ML seq-str, BA, BA species-tree) (Fig. 2A,C,D and Fig. 3A,B). The only slight exception is the ITS2 gene tree (Fig. 2B) where *Saccostrea* and Ostreinae were not reciprocally resolved. This is likely due to the low performance of the ITS2 in reconstructing phylogenetic relationships above the genus level as already pointed out in other bivalves families [28] and as indicated by the fact that when ITS2 sequences are combined with the slower evolving 28S sequences (which are linked on the same ribosomal genes cluster as the ITS2). Saccostreinae and Ostreinae are recovered as reciprocally monophyletic (Fig. 2D). A representative of *Striostrea* is available only for ITS2 and its position is unresolved (Fig. 2B). The Crassostreinae exclusive of *Saccostrea*, were supported by all our phylogenetic analyses (Figs. 2 and 3). Interestingly, the Crassostreinae ITS2 rRNA gene invariantly showed a specific sequence-structure landmark: a CBC in the basal portion of the DI ostreid consensus motif, which changed the triplet 5′-CGG/CCG-3′ to 5′-CGA/UCG-3′ (Fig. 1B). These results are in agreement with previous studies, based on 28S, 18S, 16S, COI and complete mitochondrial genome data, that found a paraphyletic arrangements of the two crassostrenids genera *Crassostraea* and *Saccostrea*, which did not form a monophyletic group but rather two monophyletic clades with high phylogenetic distance [11], [12], [19], [23], [26]. Concerning the relationships between Lophinae and Ostreinae, our results are in agreement with previous molecular studies. In particular, although Lophinae and Ostreinae taxa formed a single clade, the lack of their reciprocal monophly was recovered by [8], [12], [23] based on 16S and 28S DNA sequences data. Moreover, at any locus (16S, COI, ITS2, 28S) the average genetic distance between Lophinae and Ostreinae taxa is similar or lower than genetic distances observed within either *Crassostrea* or *Saccostrea* lineages (this study, [8], [60], [61]).

Overall, available molecular data suggest that the three main clades of Ostreidae are strongly supported and we propose to recognize them taxonomically as three subfamilies: (i) Crassostreinae, including the genus *Crassostrea* and coincident with the former tribe Crassostreini Scarlato and Starobogatov, 1979; (ii) Saccostreinae Salvi, Macali & Mariottini **subfam. nov.** urn:lsid:zoobank.org:act:C540CB87-26F1-46DC-9AC2-B98726B07519, including the genus *Saccostrea* and tentatively *Striostrea* (in this case the name Striostreinae Harry, 1985 would apply); and (iii) Ostreinae, grouping taxa previously referred to Ostreinae and Lophinae. This classification is partially supported also by non-molecular data. Indeed, although morphological characters suggest a closer affinity between *Crassostrea* and *Saccostrea*, they also provide evidence of their reciprocal distinctiveness [1], [4], [24]. Moreover, Lophinae taxa, which are included in the Ostreinae subfamily based on molecular data, share with Ostreinae a significant life history trait such as a brooding reproductive strategy, in contrast with remaining oysters which are broadcast spawners [12].

The inter-relationships between these three oyster lineages inferred in this study and in previous studies are not completely clear. We found a closer relationships between Saccostreinae and Ostreinae/Lophinae in most phylogenetic trees based on mitochondrial and nuclear gene data either alone or combined, although often with low statistical support (Fig. 2A,B,C,D and Fig. 3B). This result is in agreement with the concatenated analysis of 12 mitochondrial (protein coding) genes performed by [26] employing species of the genera *Crassostrea*, *Saccostrea*, and *Ostrea*. On the other hand, the ML trees based on 28S data and on combined mitochondrial and nuclear data (Figure S2 and Fig. 3B) as well as the COI tree showed in [11] would suggest a closer relationships between *Crassostrea* and *Ostrea*. These two genera also show a similar mitochondrial gene order compared to *Saccostrea* [26].

Below the subfamily level, our results suggest that several genera need taxonomic revision. The genus *Crassostrea* includes two highly differentiated lineages grouping the Asian species of the Indo-Pacific (*C. ariakensis*, *C. belcheri*, *C. gigas*, *C. hongkongensis*, *C. nippona*, *C. sikamea*) and the American species of the Atlantic Ocean (*C. gasar≡C. brasiliana*, *C. rhizophorae*, *C. virginica*). These two highly divergent clades are recovered with high support in all our phylogenetic analyses (Figs. 2 and 3). Furthermore, these two *Crassostrea* clades are diagnosed by three landmarks in the ITS2 rRNA secondary structure: (i) an A instead of a pyrimidine in the conserved single mismatch of DII in the Indopacific clade (*C. ariakensis*, *C. gigas*, *C. hongkongensis*, *C. nippona*), and (ii) a G located 3′ next to the lower quadruplet motif of the Basal STEM in the Atlantic ones (*C. gasar≡C. brasiliana*, *C. rhizophorae*, *C. virginica*) (Fig. 1B). Multiple lines of evidence indicate that these two groups should better be designed as distinct genera: (i) they form two highly supported monophyletic clades in all phylogenetic studies (this study, and reference herein); (ii) they have a strictly allopatric distribution in different oceans; (iii) the genetic divergence between Asian and American *Crassostrea* at mitochondrial and nuclear genes (16S, COI, ITS2, 28S, 18S) is similar or even higher than genetic distance observed between genera belonging to different subfamilies, i.e. between Ostreinae and Lophinae genera and between them and the genus *Saccostrea* (this study, [8], [60], [61]); (iv) according to divergent time estimates based on mitogenome data, the divergence among Asian and American Crasostrea is as ancient as 83 million years [36]; (v) Asian species have duplicated mitochondrial genes (*trnM*, *trnK*, *trnQ* and *rrnS*) compared with the American species and show an unusually high conservation of mitochondrial gene order that is very different from American species [36]; (vi) in the nuclear genome, karyological difference in size and shape of the rDNA-bearing chromosome (the chromosome where the major ribosomal RNA genes are located) clearly and consistently divide Asian and American species [62]. Bringing together this compelling molecular and biogeographical evidence we suggest the American Atlantic species to be assigned to the genus *Crassostrea* and the Asian Pacific species to a new genus. As no previous name is available, we propose the name *Magallana* Salvi, Macali & Mariottini **gen. nov.** urn:lsid:zoobank.org:act:889C5891-3D22-4AB1-BA01-4EF5D20EE45A in honour of the Portuguese explorer Fernão de Magalhães (Ferdinand Magellan), who crossed the Pacific Ocean in the first circumnavigation of the Earth. The type species of *Magallana* **gen. nov.** is *Crassostrea gigas* (Thünberg, 1793) [ = *Magallana gigas* (Thünberg, 1793) **comb. nov.**], which has been recently re-described by [63] to whom we refer for diagnosis and description. The *Magallana* **gen. nov.** includes all the Asian Pacific species currently accepted as: *C. ariakensis* (Fujita, 1913), *C. belcheri* (G.B. Sowerby II, 1871) [*C. gryphoides* (Newton & Smith, 1912) according to [64] and [1] is a synonym of *C. belcheri*], *C. bilineata* (Röding, 1798) [*C. iredalei* (Faustino, 1932) and *C. madresensis* (Preston, 1916) according to [64] and [1] are synonyms of *C. bilineata*], *C. dactylena* (Iredale, 1939), *C. gigas* [*C. angulata* (Lamarck, 1819) according to [1] is a synonym of *C. gigas*], *C. hongkongensis* Lam & Morton, 2003, *C. nippona* (Seki, 1934), *C. rivularis* (Gould, 1861), and *C. sikamea* (Amemiya, 1928).

The genus *Dendostrea* is paraphyletic relative to the genera *Lophia* and *Alectryonella* in all the phylogenetic trees (Figs. 2 and 3) in agreement with previous molecular studies [8], [12], [23] but not with morphological analysis of [4]. However, as suggested by [8] the morphological similarities between *Dendostrea* species may reflect convergent evolution due to shared ecological preferences rather than phylogenetic affinity. As discussed in the first section, phylogenetic trees and genetic distance analyses suggest that the Ostreinae genera *Ostreola*, and *Cryptostrea* are likely all synonyms of *Ostrea* according to [64] and the following clades may represent a single taxon each: (*Ostrea edulis, O. angasi*), (*Ostrea equestris, O. aupouria, O. stentina, O. spreta*), (*Ostrea conchaphila,O. lurida*), and (*Cryptostrea permollis, Ostrea puelchana*). However, the use of phylogeny and genetic divergence as sole information for defining species is problematic (e.g. [65], [66]), therefore a through integrative approach [67]–[69] is required to reach firm conclusion on *Ostrea* taxonomy.

## Conclusions

In this study, the rapidly-evolving ITS2 rRNA gene was analysed for the first time in the phylogenetic and taxonomic framework of the Ostreidae. The relatively low intraspecific divergence displayed by oyster ITS2 sequences compared to the high inter-specific differentiation observed among congeneric species, corroborates the utility of the ITS2 as a DNA barcode for their identification, echoing previous studies on other bivalve families [27], [28]. On the other hand, the high rate of molecular evolution of this marker may explain the drop of phylogenetic resolution above the genus level observed in the Ostreidae dataset such as in the case of Veneridae [28]. Despite the extensive length variation and divergence shown by ostreid ITS2 sequences, the combined analysis of the ITS2 sequence-structure allowed a straightforward homology assessment during multiple sequence alignment. Moreover, in agreement with previous simulation studies, the use of a combined model of rRNA sequence-structure evolution in a Maximum-Likelihood framework, improved accuracy and nodal support of phylogenetic trees (Fig. 1A,B; [29]). Building on this results and on previous studies [27]–[29], [31], [33], [70]–[73], the implementation of rRNA sequence-structure models is recommended for accurate phylogenetic estimates.

The multi-locus approach employed in this study allowed a robust inference of the phylogeny of Ostreidae. Research of the last decades has long-established that rely on single gene tree to infer species relationships is problematic because incongruence across gene phylogenies and species phylogeny are expected due to several factors including the stochastic sorting of lineages and among-genes variation both in molecular rates of evolution and in the amount of phylogenetic information [74]–[76]. In the multi-locus approach the information of single gene sequence datasets is combined to infer the species phylogeny providing a more accurate estimate of species relationships, especially when using models that take into account the stochastic sorting of lineages in the estimation of species trees [77], [78]. We found few differences in topology at the main nodes between the 16S, ITS2, COI and 28S gene trees (Fig. 1 A,B; Figure S1 and S2) and among them and the species trees (Fig. 2). The tree based on multiple loci consistently support the three main lineages of oysters Crassotreinae, Saccostreinae, and Ostreinae either when mitochondrial and nuclear loci are analysed independently (Fig. 1C,D) or combined (2A,B). The accuracy of the inferred species phylogeny is further supported by the stability of the main clades obtained under different phylogenetic methods (ML and BA) and under different multi-locus approaches (concatenation and coalescent-based approaches). Therefore, in contrast to the controversial information obtained from morphological characters, molecular data provide a well-supported phylogenetic and systematic framework for Ostreidae suggesting that the subfamilies, Ostreinae Saccostreinae **subfam. nov.** and Crassotreinae, and the further subdivision of the latter in the genera *Crassostrea* and *Magallana* **gen. nov.**, better represent the hierarchical relationships of oysters along their evolutionary history.

## Supporting Information

Figure S1 **Neighbor-Joining phylogenetic tree of the Ostreidae derived from 783 COI sequences obtained from GenBank.** The analysis was conducted in MEGA6 [Tamura et al., 2013. Mol Biol Evol 30: 2725–2729] based on the Kimura 2-parameter distances and using *Hyotissa* hyotis, *H. imbricata* and *Neopycnodonte cochlear* as outgroup. Genbank accession numbers are provided after species names; bootstrap support over 1000 replicates is reported for the three main lineages: Crassostreinae, in violet; Saccostreinae, in green; and Ostreinae, in blue. * indicate sequences selected for the combined analyses and ** indicate sequences added for the species-tree analysis.
(PDF)

Figure S2 **Maximum-Likelihood phylogenetic tree of the Ostreidae derived from 42 sequences of 28S obtained from GenBank.** The analysis was conducted in MEGA6 [Tamura et al., 2013. Mol Biol Evol 30: 2725–2729] based on the Tamura-Nei model with a discrete Gamma distribution, and using *Hyotissa* hyotis, *H. imbricata* and *Neopycnodonte cochlear* as outgroup. Genbank accession numbers are provided after species names; bootstrap support (>70) over 100 replicates is reported. The three main lineages are coloured as follows: Crassostreinae in violet; Saccostreinae in green; and Ostreinae in blue. * indicate sequences selected for the combined analyses and ** indicate sequences added for the species-tree analysis.
(PDF)
