# Correction: Molecular Phylogenetics and Systematics of the Bivalve Family Ostreidae Based on rRNA Sequence-Structure Models and Multilocus Species Tree

**DOI:** 10.1371/journal.pone.0116014

**Published:** 2014-12-15

**Authors:** The PLOS ONE Staff

There is an error in the seventh sentence of the Abstract. The subfamily name is misspelled. The correct sentence is: Therefore, the subfamily Crassostreinae (including *Crassostrea*), Saccostreinae **subfam. nov.** (including *Saccostrea* and tentatively *Striostrea*) and Ostreinae (including Ostreinae and Lophinae taxa) are recognized.

There is a typographical error in the second sentence of the second paragraph in the “Dataset, DNA extraction, amplification and sequencing” section of the Methods. The correct sentence is: It is worth mentioning that, despite some oysters show a split of the 16S rRNA gene into two segments, e.g. in the Asian *Crassostrea*, *C.* *virginica*, *Saccostrea mordax*, and *Ostrea edulis* [26], [36], our target 16S fragment (3′ half portion of the 16S rRNA gene corresponding to domains IV and V) is entirely located in the second segment.
