# Supplementary material for: Molecular Phylogenetics and Systematics of the Bivalve Family Ostreidae Based on rRNA Sequence-Structure Models and Multilocus Species Tree
Source: PLoS One. 2014 Sep 24;9(9):e108696. doi: 10.1371/journal.pone.0108696 (PMC4177229; doi:10.1371/journal.pone.0108696)

Supplementary Figure S1. Neighbor-Joining phylogenetic tree of the Ostreidae derived from 783 COI sequences (548 positions) obtained from GenBank. The analysis was conducted in MEGA6 [Tamura et al., 2013. *Mol Biol Evol* 30: 2725-2729] based on the Kimura 2-parameter distances and using *Hyotissa hyotis*, *H. imbricata* and *Neopycnodonte cochlear* as outgroup. Genbank accession numbers are provided after species names; bootstrap support over 1000 replicates is reported for the three main lineages: Crassostreinae, in violet; Saccostreinae, in green; and Ostreinae, in blue. * indicate sequences selected for the multi-locus combined analyses and ** indicate sequences added for the species-tree analysis.

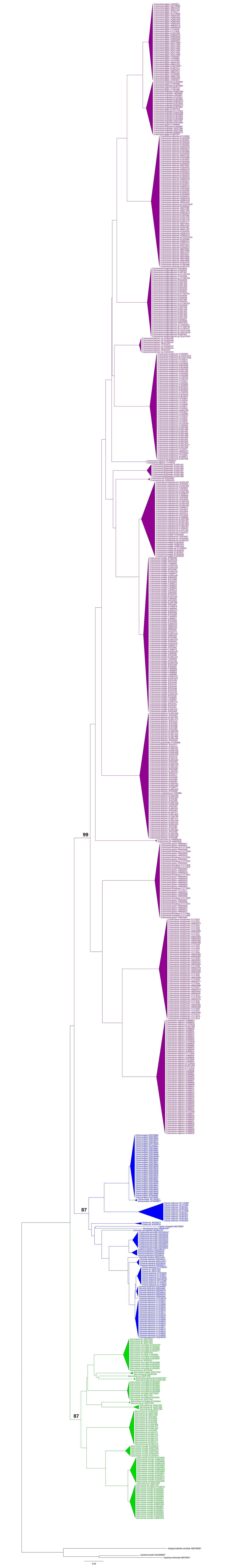

Supplement: Figure S1 — Neighbor-Joining phylogenetic tree of the Ostreidae derived from 783 COI sequences obtained from GenBank. The analysis was conducted in MEGA6 [Tamura et al., 2013. Mol Biol Evol 30: 2725–2729] based on the Kimura 2-parameter distances and using Hyotissa hyotis, H. imbricata and Neopycnodonte cochlear as outgroup. Genbank accession numbers are provided after species names; bootstrap support over 1000 replicates is reported for the three main lineages: Crassostreinae, in violet; Saccostreinae, in green; and Ostreinae, in blue. * indicate sequences selected for the combined analyses and ** indicate sequences added for the species-tree analysis. (PDF) [file pone.0108696.s001.pdf]
